# The utility of ctDNA in detecting minimal residual disease following curative surgery in colorectal cancer: a systematic review and meta-analysis

**DOI:** 10.1038/s41416-022-02017-9

**Published:** 2022-11-08

**Authors:** Lucy G. Faulkner, Lynne M. Howells, Coral Pepper, Jacqueline A. Shaw, Anne L. Thomas

**Affiliations:** 1 Leicester Cancer Research Centre, Department of Genetics and Genome Biology, University of Leicester, Leicester Royal Infirmary, Leicester, LE2 7LX UK; 2 Department of Library and Information Services, University Hospitals of Leicester NHS Trust, Leicester, LE1 5WW UK

**Keywords:** Prognostic markers, Disease-free survival, Tumour biomarkers, Colorectal cancer

## Abstract

**Introduction:**

Colorectal cancer is the fourth most common cancer in the UK. There remains a need for improved risk stratification following curative resection. Circulating-tumour DNA (ctDNA) has gained particular interest as a cancer biomarker in recent years. We performed a systematic review to assess the utility of ctDNA in identifying minimal residual disease in colorectal cancer.

**Methods:**

Studies were included if ctDNA was measured following curative surgery and long-term outcomes were assessed. Studies were excluded if the manuscript could not be obtained from the British Library or were not available in English.

**Results:**

Thirty-seven studies met the inclusion criteria, involving 3002 patients. Hazard ratios (HRs) for progression-free survival (PFS) were available in 21 studies. A meta-analysis using a random effects model demonstrated poorer PFS associated with ctDNA detection at the first liquid biopsy post-surgery [HR: 6.92 CI: 4.49–10.64 *p* < 0.00001]. This effect was also seen in subgroup analysis by disease extent, adjuvant chemotherapy and assay type.

**Discussion:**

Here we demonstrate that ctDNA detection post-surgery is associated with a greater propensity to disease relapse and is an independent indicator of poor prognosis. Prior to incorporation into clinical practice, consensus around timing of measurements and assay methodology are critical.

**Protocol registration:**

The protocol for this review is registered on PROSPERO (CRD42021261569).

## Introduction

Colorectal cancer is the fourth most common cancer in the UK. In the last few decades, there has been a steady increase in incidence within developed countries, with the UK now seeing around 35,000 cases a year. Mortality increases with stage, and collectively, colorectal cancer is responsible for 10% of all cancer deaths in the UK [1, 2]. Definitive treatment involves surgical resection, aided by perioperative chemotherapy [3]. Identification of patients who will benefit from adjuvant chemotherapy remains a dilemma, particularly in stage II disease [4].

Minimal residual disease (MRD) is defined as microscopic neoplastic material remaining after curative treatment not detectable clinically [5], and thus holds the potential to precipitate disease relapse. Recently, there has been much interest in the ability of circulating tumour DNA (ctDNA) for detection of MRD and prognostication following curative treatment including surgical resection and radical chemoradiotherapy.

This ctDNA is released from dying cancer cells and is found in varying proportions amongst cell-free DNA (cfDNA) released following the death of normal circulating blood cells. It is released during apoptosis and necrosis and has a half-life of around 2 h [6, 7]. The concept of utilising circulating tumour-derived material to provide diagnostic information on cancer has been coined ‘liquid biopsy’ [8]. The liquid biopsy has many potential advantages over the traditional surgical biopsy. It is minimally invasive and amenable to repeat measurements over time. Liquid biopsies could overcome the spatial limitation of tissue biopsies with variations in genetic profiles seen within the tumour itself and between metastases [9, 10], and could theoretically provide a more complete picture of the molecular profile.

Despite the promise ctDNA holds, there are still a number of limitations. ctDNA comprises only a minor proportion of total cfDNA, thus sensitive methods are required for detection [11]. Clonal haematopoiesis of indeterminate potential (CHIP) are non-tumour derived somatic mutations in haemopoietic cells which can bring the possibility of false positive results [8]. There are two main approaches to ctDNA analysis. Initially measurement relied on PCR-based techniques targeting a few loci. This focused approach is quick and relatively inexpensive. The ability to detect very low variant allele frequencies (VAF) brings high sensitivity, with digital-PCR and BEAMing techniques able to detect VAFs as low as 0.01% [12]. However, PCR-based techniques rely on prior knowledge of the genetic profile of the cancer and have limited capabilities for multiplexing [7]. More recently, the development of next generation sequencing (NGS) has enabled analysis of a much wider panel of target genes and enables screening for unknown variants [7, 13]. There is a growing interest in the characteristics of ctDNA beyond the somatic mutations, including methylation and fragmentation patterns [14].

At present there remains an urgent clinical need for a better post-operative risk stratification paradigm in colorectal cancer, with current tumour markers lacking sensitivity and rising late following disease recurrence [15, 16]. It has been acknowledged that ctDNA holds great potential for this application, evidenced in a number of other primary cancer sites including pancreatic [17], lung [18] and breast [19] cancer, yet there remains little consensus on the validity of this approach in colorectal cancer compounded by a lack of systematic evidence. This systematic review examines the utility of post-surgical ctDNA for detecting MRD following curative surgery in colorectal cancer, and compares study methodologies to facilitate recommendations for optimal study design for future research and integration into clinical practice.

## Methods

### Search strategy and study selection

An electronic search of MEDLINE, EMBASE and the Cochrane Library was conducted in July 2021. There was no restriction by language and no limits were applied to the search. The search strategy is available in Supplementary Material. The protocol was registered on PROSPERO (CRD42021261569). Study selection, data extraction and quality assessment were performed in duplicate with two authors (LF and LH) working independently. Disagreements were resolved by discussion between authors. All abstracts identified by the search strategy were screened and potentially eligible manuscripts were then reviewed. Study authors were contacted where relevant outcome data was missing from manuscripts.

In order for inclusion, studies had to meet the following prespecified criteria: [1] Participants had to be diagnosed with colorectal cancer and undergoing curative surgical resection. [2] Post-operative ctDNA measurement was performed. [3] Participant follow-up had to be such that long-term outcomes could be assessed.

Surgical procedures on primary colorectal cancer, local recurrences and metastasectomies were included, provided they were carried out with curative intent. The post-operative ctDNA measurement could be carried out at any timepoint post-operatively provided this measurement was then correlated with long-term outcomes. Any length of follow-up were considered provided time to relapse or death were measured during this time. Studies were excluded if the manuscript could not be obtained from the British Library or were not available in English. Unpublished work was not included. We accepted any study design, however case report and reviews were not included. There was no restriction by publication date or sample size.

### Data extraction

Data extraction was conducted in accordance with the following criteria: study characteristics (author, date of publication, country); study design (sample size, prospective/retrospective, follow-up time); participant baseline characteristics (age, gender, site, stage, neoadjuvant/adjuvant chemotherapy); ctDNA methodology (timing of samples, assay, gene panel, limit of detection, cut-off value).

At present there is no gold-standard method of detection of MRD, so long-term outcomes were used as surrogate markers, with the hypothesis that those with undetected residual disease will have a higher propensity to relapse. The outcomes collected were the proportion of subjects classified as ctDNA-positive at the first liquid biopsy after surgery, the proportion of participants who relapsed in each group, median progression-free survival (PFS), median overall survival (OS) and the corresponding hazard ratios (HRs) confidence intervals and *p* values.

### Quality assessment

A quality assessment form was designed by considering relevant aspects from each domain in the ROBINS-I risk of bias tool [20]. This generated a ten-point scale. The mapping of each question to the domains of bias according to the ROBINS-I tool are shown in Supplementary Table 1. For each criterion, studies could be graded as ‘low risk’, ‘high risk’ or ‘unsure’. Each study was then scored out of 11, with the final score incorporating study timeline (i.e. prospective/retrospective). We also collected information on centre number, sample size and statistical adjustment.

Both the data extraction form and quality assessment form were pre-piloted and can be found in the supplementary material.

### Data synthesis

A meta-analysis was conducted combining the HRs for PFS of ctDNA-positive vs ctDNA-negative groups. HR were pooled by inverse variance using the overall estimated HR and standard error of individual studies, either from data presented in the manuscripts or from a Cox proportional-hazards model from individual participant data available provided as a supplement or obtained directly from the study authors. Heterogeneity was quantified with the *I*^2^ statistical test and a random-effect model was used in the presence of significant heterogeneity (*p* < 0.05 or *I*^2^ ≥ 50%). Subgroup analysis was performed according to disease extent (primary resection vs metastasectomy), adjuvant chemotherapy and assay type (NGS vs PCR), as pre-planned. Results were displayed in Forest plots. Publication bias was assessed by Funnel plot to assess for asymmetry.

This review adheres to the Preferred Reporting Items for Systematic Reviews and Meta-analyses (PRISMA) guidelines [21] and the Meta-analysis of Observational Studies in Epidemiology (MOOSE) guidelines [22]. Statistical analysis was performed on Review Manager (RevMan) Version 5.4, The Cochrane Collaboration (2020).

### Reporting summary

Further information on research design is available in the Nature Research Reporting Summary linked to this article.

## Results

### Search results

**Fig. 1:**
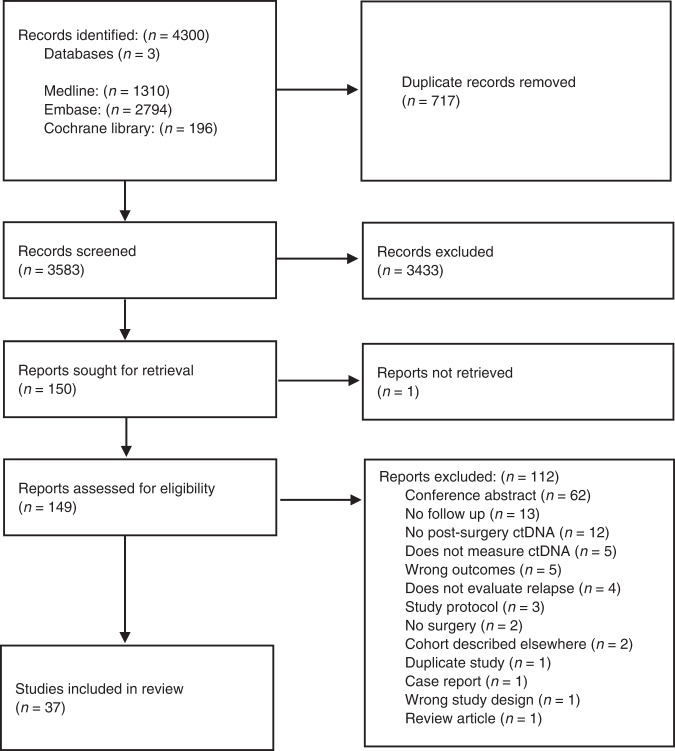
PRISMA flow diagram. Flow diagram describing the study selection process and number of studies at each stage according to the Preferred Reporting Items for Systematic Reviews and Meta-analyses (PRISMA) guidelines.

The search identified 3581 papers, after removal of duplicates. Full-text screening was performed for 147 studies, of which 37 studies were included involving 3002 patients (Fig. 1) [23–59]. Details of the key excluded studies can be found in Supplementary Table 2.

### Included studies

Included studies incorporated all stages of colorectal cancer (I–IV), with six specific to rectal cancer. On average, 42.2% of patients had rectal cancer and 34.8% exhibited right-sided disease. Articles were published between 1993 and 2021 and were conducted in continents including North America, Europe, Asia and Australasia. Surgical procedures included removal of the primary cancer, local recurrence and metastasectomy. Nine papers addressed metastasectomy alone (liver, peritoneal or lung) with a further two including metastasectomy sub-groups. The median age ranged from 55 to 73 and the proportion of male participants ranged from 33-90%, mean 53.2% (Table 1). Out of 37 papers, only 16 (43.2%) reported the proportion of patients who received neoadjuvant chemotherapy (range = 0–100% participants, mean: 43.6%), and 26 (70.3%) the proportion of patients receiving adjuvant chemotherapy (range: 0–100%, mean: 63.5%). The most common regimen was 5FU-based, either alone or in combination with oxaliplatin. The median follow-up time of the study ranged from 11.7 months to 6.6 years (median 26.2). Post-operative monitoring protocols were described in 23 (62.2%) studies, consisting of physical examination, laboratory tumour markers (CEA, CA19.9) and radiology (Table 1).

**Table 1:** Study characteristics.

Author	Year	Journal	Country	Sample size		Follow-up (months)		Gender (% male)	Age (median)	Cancer stage	Cancer site	Neoadjuvant chemotherapy (%)	Adjuvant chemotherapy (%)	Reference
						Median	Range							
Allegretti et al.	2020	Journal of Experimental and Clinical Research	Italy	10	11.7		6–40	47.2	Not stated	Non-metastatic	Colorectal	0	Not stated	[23]
Beagan et al.	2020	Journal of Clinical Medicine	Netherlands	24	Not stated		Max 25	58.3	66.5	IV	Metastases (P)	0	37.5	[24]
Benešová et al.	2019	World Journal of Gastroenterology	Czech Republic	26	Not stated		Not stated	48.4	63.6^a^	IV	Metastases (H + L)	Not stated	Not stated	[25]
Boysen et al.	2020	Acta Oncologica	Denmark	35	21		Not stated	51.4	70.5	IV	Metastases (H + L)	Not stated	74.3	[26]
Carpinetti et al.	2015	Oncotarget	Brazil	3	Not stated		Not stated	33.3	Not stated	II–III	Rectal	100	0	[27]
Chen et al.	2021	Journal of Hematology and Oncology	China	240	27.4		26.2–28.5	56.7	60	II–III	Colon	0	72.5	[28]
Diehl et al.	2008	Nature Medicine	USA	18	Not stated		2–56 days	44.4	59.8^a^	II–IV	Colon + metastases (H + L)	Not stated	61.1	[29]
He et al.	2020	Cancer Management and Research	China	19	Not stated		14–18	70	55^a^	IV	Metastases (H)	55	80	[30]
Huang et al.	2019	Cancer Biomarkers	China	41	30		7–146 weeks	53.5	58	I–IV	Colorectal	Not stated	67.4	[31]
Ji et al.	2021	Genomics	China	32	Not stated		Not stated	59.4	58.7^a^	0–III	Rectal	100	Not stated	[32]
Jin et al.	2021	PNAS	China	73	Not stated		36–50 months	63	67	I–IV	Colorectal	83.6	84.9	[33]
Khakoo et al.	2020	Clinical Cancer Research	UK	23	26.4		(IQR 19.7–31.3)	56.5	50 (+) 59 (−)	I–III	Rectal	100	91.3	[34]
Lee et al.	2021	Cancers	South Korea	53	Not stated		Not stated	63.8	56	III–IV	Metastases (H/L/P)	47.2	Not stated	[35]
Leon Arellano et al.	2020	Disease Markers	Spain	10	26		25–29	30	67.5	II–IV	Colon	0	Not stated	[36]
Levy et al.	2012	Anticancer Research	Czech Republic	4	22.5		12–29	50	65	II–IV	Colorectal	25	75	[37]
Lindforss et al.	2005	Anticancer Research	Sweden	24	35		5–52	37.5	72	I–III	Colorectal	Not stated	Not stated	[38]
López-Rojo et al.	2020	Therapeutic Advances in Medical Oncology	Spain	9	28.5		8–41	36.3	56.9^a^	IV	Metastases (P)	Not stated	100	[39]
Mason et al.	2021	Journal of the American College of Surgeons	USA	63	30		9–53	50.8	55	IV	Metastases (H)	87.3	58.7	[40]
Murahashi et al.	2020	British Journal of Cancer	Japan	59	Not stated		Not stated	79.7	60	II–III	Rectal	100	Not stated	[41]
Murray et al.	2018	Journal of Cancer Research and Clinical Oncology	Australia	172	23.2		(IQR 14.3–29.5)	61	65.5^a^	III–IV	Colon + metastases	Not stated	Not stated	[42]
Ng et al.	2017	Scientific Reports	Singapore	10	965 days		786–1253 days	90	65.3	II–III	Colorectal	Not stated	Not stated	[43]
Parikh et al.	2021	Clinical Cancer Research	USA	70	632.5 days		33–246 days	60.7	60	I–IV	Colorectal	45.2	54.8	[44]
Reinert et al.	2019	JAMA Oncology	Denmark	125	12.5		1.4–38.5	56.9	69.9	I–III	Colorectal	Not stated	61.6	[45]
Ryan et al.	2003	Gut	Netherlands	85	28		6–72	60.6	66	Dukes A–C	Colorectal	Not stated	56.4	[46]
Scøhler et al.	2017	Clinical Cancer Research	Denmark	44 (Cohort 1: 21 Cohort 2:23)	Not stated		8 days–36 months	64.4	65	Cohort 1: I–III Cohort 2: IV	Colorectal + metastases (H)	Not stated	34.1	[47]
Suzuki et al.	2020	Oncotarget	Japan	44	366 days		Not stated	58.4	71	II–III	Colorectal	Not stated	36.4	[48]
Taieb et al.	2021	Clinical Cancer Research	France	1017	6.6 years		(95% CI 6.5–6.8)	56.6	64.4	III	Colon	Not stated	100	[49]
Tanaka et al.	2021	Scientific reports	Japan	11	22		15.4–23.6	36.4	69	I–III	Colorectal	Not stated	45.5	[50]
Tarazona et al.	2019	Annals of Oncology	Spain	69	24.7		1–45.2	64.9	71	I–III	Colon	0	37.3	[51]
Tie et al.	2016	Science Translational Medicine	Australia	167	27		2–52	57	65	II	Colon	0	23	[47]
Tie et al. (a)	2019	JAMA Oncology	Australia	96	28.9		11.6–46.4	51	64	III	Colon	Not stated	99	[37]
Tie et al. (b)	2019	Gut	Australia	159	24		1–55	67.3	62	II–III	Rectal	100	64.2	[52]
Tie et al.	2021	PLoS Medicine	Australia	38	50.5		5–82	71.4	64	IV	Metastases (H)	42.6	77.8	[31]
Yamada et al.	2016	Cancer Science	Japan	7	Not stated		Not stated	71.4	73	III–IV	Metastases (H + L)	14.3	Not stated	[53]
Zhou et al.	2016	PLoS ONE	China	5	46		32–47	33.3	64	I–III	Colorectal	0	66.7	[54]
Zhou et al.	2021	Clinical Cancer Research	China	89	18.8		3.1–21.3	65.2	60	II–III	Rectal	99	80.6	[55]
Zou et al.	2020	Carcinogenesis	New Zealand	28	510.5 days		98–692 days	48.3	65	II–IV	Colorectal + metastases (H + L)	Not stated	Not stated	[56]

Table shows study characteristics and participant characteristics of the included studies.

*IQR* interquartile range, metastases site: *H* hepatic, *L* lung, *P* peritoneal.

^a^Age presented as mean.

Timing of the first post-operative ctDNA measurement varied from the day of surgery to 13 months post-surgery. PCR-based methods were used in 19 (51%) studies and 15 (31%) used NGS, with 3 studies monitoring epigenetic changes. Fourteen (38%) reported a limit of detection (LoD) of the assay and 31 (86%) specified a cut-off level to establish ctDNA positivity. There was little consensus on the gene panel breadth, with the number of genes evaluated ranging from 1 to 1021. In 16 studies, the mutations evaluated in ctDNA were based on those previously identified in tissue (15) or plasma (1). Within these, the size of the gene panel evaluated in the tumour ranged from 4 genes to whole-genome sequencing (WGS). ctDNA was also measured pre-operatively in 32 (86%) of the studies (Table 2).

**Table 2:** Methodology.

Author, Year	Timing of post-op liquid biopsy	Limit of detection (VAF)	Cut-off level	Method of detection	Gene panel	Number of genes
Allegretti, 2020	3 months	≥0.2%	Any mutations detected	NextSeq Digital PCR plus validation with dPCR	TruSight Tumour panel	15
Beagan, 2020	2 weeks–3 months	Not stated	Not stated	ddPCR	Variants in metastases VAF ≥ 3%	(48)
Benešová, 2019	1 week	0.03–1%	Not stated	PCR and DCE	KRAS, TP53, APC, PIK3CA, BRAF, CTNNB1	6
Boysen, 2020	2 weeks	0.10%	Any mutations detected	ddPCR and MassARRAY	UltraSEEK MA Colon Panel	5
Carpinetti, 2015	Not stated	1 amplifiable copy/mL plasma	3 positive droplets from 10 to 15,000 droplets	Taqman assays	Chromosomal rearrangements from WGS of tumour	(WGS)
Chen, 2021	3–7 days	Not stated	>5% of total tracking variants	Geneseeq Prime	Geneseeq Prime™ 425-gene panel	425
Diehl, 2008	1 day	Not stated	Fraction of beads bound to mutant fragments higher than the negative control, mean mutant DNA fragments plus one standard deviation >1.0	BEAMing	Mutations detected in tumour FFPE sequencing	(4)
He, 2020	Within 7 days	Not stated	Not stated	Capture-based targeted deep sequencing	ColonCore panel NextSeq 500 system (Illumina, Inc.)	41
Huang, 2019	1 month	Not stated	>4 mutant reads in plasma with >1 read on each strand	Illumina NextSeq 500	85 genes	85
Ji, 2021	1 day	Not stated	TMB > 10/ctDNA—any mutations/change in TMB	Illumina HiSeq X-Ten	30 mutation signatures	30
Jin, 2021	1–14 days	0.05% tumour DNA	mqMSP assay: ΔCq value > −1. SEPT9 assay: at least one out of three qPCR replicates had a Ct value <45	Methylation-specific quantitative PCR assay (mqMSP)	Septin 9 (SEPT9) gene hypermethylated	NA
Khakoo, 2020	4–12 weeks	not stated	Two mutant-positive droplets present for at least one variant	ddPCR	1–3 variants with highest VAF in tumour	1–3 (6)
Lee, 2021	3–4 weeks	1%	VAF ≥ 1%	Ultra-deep targeted sequencing	Somatic variants identified from primary and metastatic tumour	(50)
Leon Arellano, 2020	3 months	Valid when the ACTB Ct was ≤32.1	SEPT9 Ct <45 cycles	Duplex quantitative PCR, Fast Real-Time PCR	Septin 9 (SEPT9) hypermethylated	1*
Levy, 2012	<1 week	Not stated	5% of mutated alleles	Fluorescently labelled PCR and DCE	Somatic mutations previously found in tumour	(5)
Lindforss, 2005	3 days	Not stated	Not stated	PCR	KRAS	1
López-Rojo, 2020	48 h	Not stated	Concentration compared between samples and wild-type controls using a *Z* test, *p* < 0.05 used for positivity	ddPCR	KRAS	1
Mason, 2021	Median 13 months (range 1–45 months)	0.30%	Any mutations detected	Guardant360 CDx	70 genes	70
Murahashi, 2020	12 weeks	Not stated	VAF 0.15%	Amplicon-based deep sequencing	14 genes	14
Murray, 2018	Within 12 months	Not stated	At least one PCR replicate positive for methylation	Triplex real-time qPCR assay	BCAT1 and IKZF1 methylation	2*
Ng, 2017	Within 5 days	0.05%	Positive on a one-tailed exact conditional test of the ratio of two Poisson rates to distinguish from negative controls	Multiplex-PCR amplicon sequencing	Somatic variants identified from the primary tumour	1–14 (799)
Parikh, 2021	11–148 days	Not stated	Any mutations detected	Guardant Reveal test	Not stated	Not stated
Reinert, 2019	30 days	Not stated	At least 2 variants detected	HiSeq 2500 system	16 somatic single-nucleotide variants and short indels based on WES of tumour	16 (WES)
Ryan, 2003	1 week	Not stated	Not stated	PCR	KRAS	1
Scøhler, 2017	8 days	0.50%	Any mutations detected	ddPCR	Mutations identified on WES of tumour	Mean 4.2 (WES)
Schou, 2018	3 months	170 ng/mL	Above 75th quartile	Direct fluorescent assay	Not applicable	not applicable
Suzuki, 2020	At the end of hospitalisation	0.02%	NGS: at least one mutated ctDNA PCR: one copy of mutated ctDNA	Pre op: Oncomine Pan Cancer Cell Free Assay. Post op: ddPCR	Mutations identified pre-surgery in plasma by NGS	((52))
Taieb, 2021	After surgery, before adjuvant chemotherapy	Above limit of blank	Above limit of blank	Multiplex droplet-based digital PCR (ddPCR) and NGS	WIF1 and NPY gene hypermethylation (AmpliSeq Colon and Lung Cancer Panel V2 performed in a subset of patients)	2*+22
Tanaka, 2021	1 day	0.10%	VAF 0.15%	dPCR Taqman assays	BRAF	1
Tarazona, 2019	6–8 weeks	Not stated	VAF 5%	Orthogonal droplet digital PCR	Two mutations with the highest VAF on NGS of tumour	2 (29)
Tie, 2016	4–10 weeks	Not stated	Permutation test comparing mutation frequency between samples and controls	Illumina MiSeq	Somatic mutation with highest VAF in tumour FFPE	1 (15)
Tie, 2019 (1)	4–10 weeks	Not stated	Permutation test comparing mutation frequency between samples and controls	Illumina MiSeq	Mutation with the highest VAF in tissue from surgery	1 (15)
Tie, 2019 (2)	4–10 weeks	Not stated	Permutation test comparing mutation frequency between samples and controls	Safe-SeqS and Illumina MiSeq	Somatic mutation with the highest VAF in tumour tissue	1 (15)
Tie, 2021	4–10 weeks	Not stated	Permutation test comparing mutation frequency between samples and controls	Safe-SeqS	Mutation with the highest VAF in tumour tissue	1 (15)
Yamada, 2016	Within 1 month	<1.00%.	Ratio of 0.1% mutant to 99.9% wild type	Invader Plus assay with peptide nucleic acid clamping method and digital PCR	KRAS	1
Zhou, 2016	1 month	Not stated	VAF > 0	Illumina HiSeq 2500	545 genes	545
Zhou, 2021	Within 1 month	Not stated	At least one mutation in ctDNA also detected in tissue	HiSeq 3000 Sequencing System (Illumina)	1021 genes	1021
Zou, 2020	12 weeks	Not stated	VAF 1%	ddPCR	Somatic mutations from targeted sequencing of FFPE slides	2 (71)

Table shows analysis methods of measurement of ctDNA in post-operative blood samples. *Gene methylation; () genes evaluated in tumour tissue; (()) genes evaluated in pre-op blood samples.

*DCE* denaturing capillary electrophoresis, *VAF* variant allele frequency, *ddPCR* digital droplet PCR, *NGS* next-generation sequencing, *PCR* polymerase chain reaction, *qPCR* quantitative PCR, *VAF* variant allele frequency, *WES* whole-exome sequencing, *WGS* whole-genome sequencing.

### Association of ctDNA with PFS

The proportion of participants classified as ctDNA-positive at the first liquid biopsy after surgery ranged from 0 to 90.9% (median 20%). In 3 studies, no patients had detectable ctDNA at the first liquid biopsy after surgery [23–25]. The proportion of patients who relapsed during follow-up was consistently higher in ctDNA positive participants concurrent with shorter median PFS (Table 3). Time-to-event analysis for PFS according to post-operative ctDNA was available for 21 studies including 2645 participants. This included outcomes calculated from data available in the supplementary material [26] and data sent by the study authors [27]. Multivariate analysis had been performed in 15 studies and OS was assessed in 12 (Supplementary Table 3). A shorter PFS associated with ctDNA-positivity was consistently observed, with HRs varying between 1.36 and 39.9. This was statistically significant in 19 studies via univariate analysis and in all multivariate analysis (Table 3).

**Table 3:** Disease relapse.

Study		ctDNA positive *n* (%)	Relapse (%)		Median PFS (months)		Univariate					Multivariate		
			ctDNA positive	ctDNA negative	ctDNA positive	ctDNA negative	Hazard ratio	Confidence interval			*p* value	Hazard ratio	Confidence interval	*p* value
Allegretti, 2020		3 (30%)	100	0	3	Not reached	–		–	–		–	–	–
Beagan, 2020		1 (4.2%)	100	56.5	7	Not reached	–		–	–		–	–	–
Benešová, 2019		2 (7.1%)	100	58.3	6	Not stated	–		–	–		–	–	–
Boysen, 2020		5 (14.3%)	80	37.1	9	Not reached	3.36		1.03–10.94	0.03		7.48	1.47–38.36	0.02
Carpinetti, 2015		1 (33.3%)	100	0	10	Not reached	–		–	–		–	–	–
Chen, 2021		20 (8.3%)	60	0.5	Not stated	Not stated	10.98		5.31–22.72	<0.001		8.02	3.59–17.92	<0.001
							9.99**		4.40–22.69**	<0.001**		–	–	–
Diehl, 2008		10 (55.6%)	100	33.3	Not stated	Not stated	–		–	–		–	–	–
He, 2020		4 (20%)	Not stated	Not stated	5	Not reached	–		–	–		–	–	–
Huang, 2019		9 (23.1%)	33.3	3.3	Not stated	Not stated	10.767		1.1–103.8	0.04				
Ji, 2021		19 (59.4%)	38.5	10.5	Not stated	Not stated	–		–	–		–	–	–
Jin, 2021		21 (28.8%)	52.4	17.3	Not stated	Not stated	4.2		2.3–18.73	0.0005		–	–	–
							4.08*		1.26–75.05*	0.037*		–	–	–
							5.16**		2.31–29.78**	0.001**		–	–	–
	Localised	–	–	–	–	–	4.04		1.98–23.07	0.001		–	–	–
Khakoo, 2020		3 (13%)	100	0	Not stated	Not stated	39.9		4.0–399.5	0.002				
Lee, 2021		0	NA	52.8	NA	23.6	–		–	–		–	–	–
Leon Arellano, 2020		4 (40%)	100	0	Not stated	Not stated	–		–	–		–	–	–
Levy, 2012		0	NA	25	NA	23	–		–	–		–	–	–
Lindforss, 2005		8 (33.3%)	50	23.5	12	Not reached	1.77		0.47–6.62	0.396				
López-Rojo, 2020		5 (55.6%)	100	0	8.3	35.4	–		–	–		–	–	–
Mason, 2021		42 (66.7%)	26.2	Not stated	Not stated	Not stated	–		–	–		–	–	–
Murahashi, 2020		21 (35.6%)	23.8	18.8	Not stated	Not stated	20		5.6–72	<0.0001		7.7	1.6–42	0.0127
Murray, 2018		28 (16.3%)	25	11.1	Not stated	Not stated	–		–	–		3.81	1.5–9.5	0.004
Ng, 2017		0	NA	10	NA	Not stated	–		–	–		–	–	–
Parikh, 2021		17 (24.2%)	88.2	24.5	5.3	Not reached	11.2		Not stated	<0.0001		–	–	–
		–	–	–	–	–	12.03*		1.77–81.7*	<0.0001*		–	–	–
		–	–	–	–	–	7.35**		1.72–31.42**	<0.0001**		–	–	–
Reinert, 2019		10 (10.6%)	70	11.9	Not stated	Not stated	–		–	–		–	–	–
							–		–	–		4.5*	1.6–12.8*	0.004*
	Post-surgery						7.2**		2.7–19.0**	<0.001**				
	Post-ACT						17.5**		5.4–56.5**	<0.001**		–	–	–
Ryan, 2003		15 (17.6%)	60	11.4	Not stated	Not stated	–		–	–		6.37	2.26–18.0	0
Scøhler, 2017	Primary resection	6 (28.6%)	100	27	Not stated	Not stated	37.7		2–335.5	<0.001		–	–	–
	Metastasectomy	7 (30.4%)	100	50	Not stated	Not stated	4.93		1.5–15.7	0.007		–	–	–
Suzuki, 2020		6 (13.6%)	50	2.6	Not stated	Not stated	23.8		2.45–250	0.006		–	–	–
Taieb, 2021		139 (13.7%)	35	27	(66.4% at 3 years)	(76.7% at 3 years)	1.46**		1.08–1.97**	0.015**		1.55	1.13–2.12	0.006
Tanaka, 2021		10 (90.9%)	40	0	15.4	Not reached	–		–	–		–	–	–
Tarazona, 2019		14 (20.3%)	57.1	13	Not stated	Not stated	6.96		Not stated	0.0001		11.64	3.67–36.88	<0.001
Tie, 2016	All						13		6.6–27	<0.001		14	6.8–28	<0.001
	No chemotherapy	14 (7.9%)	78.6	9.8	9.9 (0% at 3 years)	Not reached (90% at 3 years)	18*		7.9–40*	<0.001*		28*	11–68*	<0.001*
	Adjuvant chemo	6 (11.5%)	Not stated	Not stated	Not stated	Not stated	11**		1.8–68**	0.001**		–	–	–
Tie, 2019 (a)	Post-surgery	20 (20.8%)	45	14.5	20.6 (47% at 3 years)	Not reached (76% at 3 years)	3.8**		2.4–21**	<0.001**		7.5**	3.5–16.1**	<0.001**
	Post-chemo	15 (17%)	66.7	17.8	(30% at 3 years)	(77% at 3 years)	6.8**		11–157**	<0.001**		–	–	–
Tie, 2019 (b)	All	19 (11.9%)	58	8.60	(33% at 3 years)	(87% at 3 years)	13		5.5–31	<0.001		6.0	2.2–16	<0.001
	No chemo						22*		4.2–110*	<0.001*		–	–	–
	Adjuvant chemo	11 (10.8%)	100	17.3	(50% at 3 years)	(85% at 3 years)	10**		3.4–29**	<0.001**		–	–	–
Tie, 2021	Post-op	21 (24.5%)	83.3	30	(69.3% at 5 years)	(16.7% at 5 years)	6.26		2.58–15.2	<0.001		3.13	1–9.82	0.05
	Post-chemo						14.94		4.94–44.7	<0.001		–	–	–
Yamada, 2016		2 (28.6%)	0	60	Not stated	7.5	–		–	–		–	–	–
Zhou, 2016		1 (20%)	25	0	Not stated	Not stated	–		–	–		–	–	–
Zhou, 2021		6 (6.7%)	100	6	Not stated	Not stated	25.3		1.475–434	<0.001		1.267	Not stated	<0.001
Zou, 2020		2 (7.14%)	100	0	11.3	62.7	–		–	–		–	–	–

Table shows the proportion of patients who were identified as ctDNA positive from the first liquid biopsy after surgery, disease relapse rate, median PFS and time-to-event analysis for PFS according to ctDNA results. Percentage PFS from Kaplan–Meier estimates.

*CI* confidence interval, *HR* hazard ratio, *PFS* progression-free survival, *NA* not applicable.

^a^Sample did not receive adjuvant chemotherapy.

^b^Sample received adjuvant chemotherapy.

### Meta-analysis of PFS according to ctDNA

**Fig. 2:**
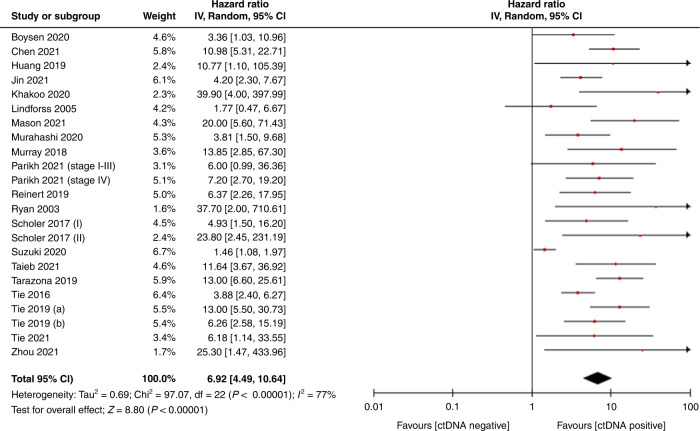
Forest plot showing meta-analysis for PFS according to post-operative ctDNA following surgery for colorectal cancer. Data displayed as HR with 95% confidence intervals on a logarithmic scale. HR hazard ratio, PFS progression-free survival, SE standard error.

**Fig. 3:**
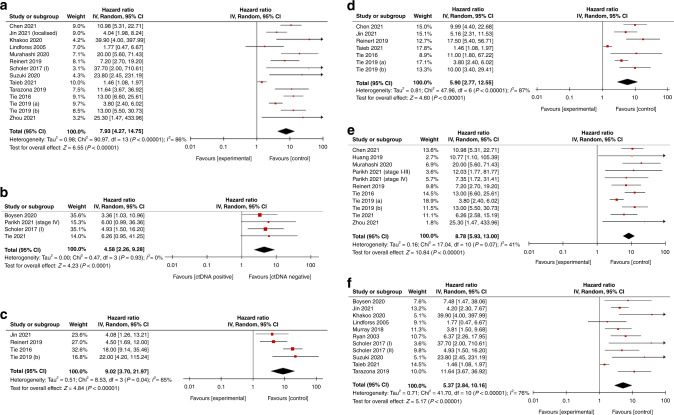
Subgroup analysis. Forest plot showing subgroup meta-analysis for PFS according to post-operative ctDNA according to disease extent, adjuvant chemotherapy and assay type: **a** resection of primary disease; **b** metastasectomy, **c** did not receive adjuvant chemotherapy; **d** received adjuvant chemotherapy; **e** NGS; **f** PCR data displayed as HR with 95% confidence intervals on a logarithmic scale. HR hazard ratio, NGS next-generation sequencing, PCR polymerase chain reaction, PFS progression-free survival.

**Fig. 4:**
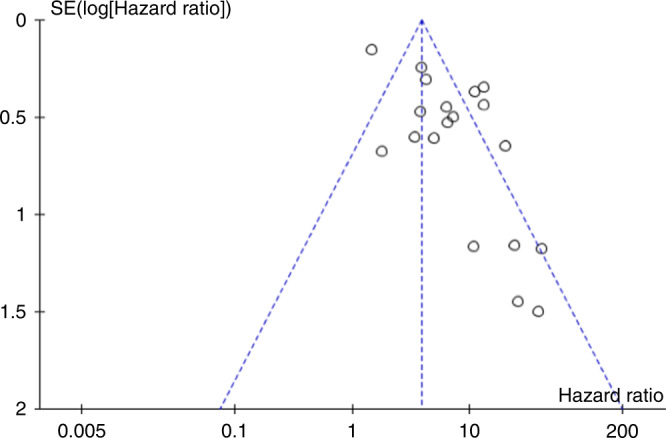
Funnel plot. Funnel plot to show effect size against standard error for HR of PFS according to ctDNA status. HR hazard ratio, PFS progression-free survival, SE standard error.

A meta-analysis confirmed poor prognosis associated with ctDNA detection post-operatively, which was found to be statistically significant [HR 6.92, CI 4.49–10.64, *p* < 0.00001] (Fig. 2). This effect was also seen in subgroup analysis according to adjuvant chemotherapy use [adjuvant chemotherapy HR 6.01, CI 2.96–12.21, *p* < 0.00001, no adjuvant chemotherapy HR 10.3, CI 6.46–16.45, *p* < 0.00001], disease extent [primary resection HR 7.93, CI 4.27–14.75, *p* < 0.00001 metastasectomy HR 5.08, CI 2.85–9.05, *p* < 0.00001] and assay type [NGS: HR 8.87, CI 5.93–13, *p* < 0.00001; PCR: HR 5.37, CI 2.84–10.16, *p* < 0.00001] (Fig. 3). A meta-analysis was also performed where multivariate analysis was available [HR 5.73, CI 3.34–9.84, *p* < 0.00001] (Supplementary Fig. 1). Statistical testing demonstrated significant heterogeneity (*p* < 0.00001) with an *I*^2^ value of 77%, hence a random effects model was used. The funnel plots of effect size (HR) plotted against standard error showed asymmetry suggestive of publication bias (Fig. 4).

### Association of ctDNA with OS

Hazard ratios comparing overall survival were available in five papers [28–32]. An association of poor prognosis with post-operative ctDNA detection was also seen on meta-analysis when comparing overall survival [HR 3.64, CI 1.63-8.12, *p* = 0.002] (Supplementary Table 3 and Supplementary Fig. 2).

### Quality assessment

The total quality assessment score of included studies ranged from 7 to 11 out of 11 (Supplementary Table 4). Patient baseline characteristics and ctDNA methodologies were generally well described. Most studies were conducted in single centres [32] and sample size calculations were rarely performed [4]. There were a number of studies with small sample sizes and inclusion of only a few participants; however, of those included in the meta-analysis the minimum sample size was 24 owing to the need for sufficient data for meaningful survival analysis in these studies.

## Discussion

In this review, we demonstrate that ctDNA detection after curative surgery in colorectal cancer is associated with shorter time to disease relapse. This relationship was consistently demonstrated across multiple studies, and here we demonstrate for the first time that this effect is statistically significant when combined through a meta-analysis. The role of ctDNA as a marker of prognosis has previously been explored in Stage IV disease; a systematic review included four studies looking at resectable disease incorporating 123 patients. They report a ‘lead time’ with ctDNA appearance and disease relapse compared to detection by imaging, but did not find a significant relationship between pre-surgery ctDNA and overall survival [33]. As far as we are aware this is the first meta-analysis combining survival analysis between ctDNA detection and long-term outcomes and is the first review examining this effect in resectable disease across all disease stages. Despite the large volume of research on this topic, there remains a lack of consensus on a number of practical aspects. This resulted in considerable variability between studies, introducing heterogeneity into the analysis and was the main limitation of this review.

Post-operative ctDNA measurement could influence clinical management at a number of points. Recognition of patients at low-risk of relapse would enable identification of individuals in whom adjuvant therapy was unnecessary, whereas ctDNA measurement after completion of adjuvant treatment could be used to determining the need for further treatment [34, 35]. ctDNA could also be incorporated into ‘watch and wait’ protocol in rectal cancer following complete response to neoadjuvant chemotherapy. Liquid biopsy could also be incorporated into the assessment of response to other modalities of curative treatments including radical radiotherapy. Additionally, ctDNA could be used to guide post-treatment surveillance through identification of patients in whom more intensive monitoring is warranted.

There was little consensus across studies regarding timing of ctDNA sampling. Three studies measured ctDNA both post-surgery and after completion of adjuvant chemotherapy, demonstrating the post-chemotherapy time-point to be a stronger predictor of prognosis [31, 36, 37]. In order to be of clinical utility, detection of MRD should be performed at a time when it is possible to influence disease management. Delay in commencing adjuvant chemotherapy beyond eight weeks is associated with worse long-term outcomes [38], meaning that post-surgical ctDNA timings will be a critical consideration when being incorporated into treatment pathways. Analysis should be performed once ctDNA from the primary tumour has been cleared from the circulation. Clearance of ctDNA following surgery was investigated by Chen et al. through serial measurement in the immediate post-operative period following resection of lung cancer; they showed that ctDNA continues to decrease until three days post-surgery and that detection past this time point correlated better with prognosis [39]. Another important consideration in assay timing is that cfDNA rises with physiological stresses, including surgery. Henriksen et al. recently investigated the sequence of cfDNA and ctDNA post-operatively in colorectal and bladder cancer; they found that short cfDNA rose and remained significantly elevated for four weeks following surgery and recommend repeat ctDNA analysis at four weeks for any patients in whom ctDNA is not detected immediately post-op [40].

Gene panel selection remains a challenge in many aspects of precision oncology. There was a wide variation in the breadth of gene panels in this review as a result of the combination of PCR and NGS-based techniques. More comprehensive gene/mutation panels will enable detection of rarer mutations [41], but bring the possibility of false positives from CHIP [8]. Some of the studies in this review investigated presence of germline mutations either by sequencing DNA from peripheral blood leucocytes or based on the ctDNA VAF.

A tumour-informed approach was adopted by 16 studies, tracking previously identified mutations. This personalised approach brings the advantage of improved specificity whilst also achieving a high sensitivity using PCR-based assays [42]. However, the need for individualised assay development will be more logistically difficult to incorporate into routine care.

An alternative approach to identifying somatic mutations is to assess epigenetic changes. Although technically more challenging to measure, methylation changes are more consistent across a cancer type and occur early in the cancer pathophysiology. Four papers in this review assessed gene methylation [29, 30, 43, 44]. Parikh et al. investigated both genetic and epigenetic changes in NGS analysis of 103 patients undergoing curative surgery for stage I–IV colorectal cancer and concluded that integrating both genetic and epigenetic changes increases sensitivity for MRD detection [44].

Assay sensitivity is of significance in the setting of MRD, where disease bulk is low. Of our included studies, Suzuki et al. report the lowest LoD of 0.02% using ddPCR [27] (Table 2). In three studies, none of the cohort had detectable ctDNA after surgery [23–25] (Table 3), yet in all three studies, a subset of patients went on to relapse which may have represented ctDNA levels below the sensitivity of these assays. The majority of studies in this review measured pre-surgical ctDNA. In three studies, detection of ctDNA pre-surgery was a requirement for inclusion in the post-operative analysis [27, 45, 46], which may serve to remove ‘non-shedders’ or ‘low shedders’, a subset of patients whose tumour does not release ctDNA.

Statistical testing showed significant heterogeneity between studies, which is likely to affect the repeatability and external validity of this review. This remains the main limitation of this review and of application to clinical practice. Clinical heterogeneity will have arisen from differences in study design. Differences in the approach to removal of CHIP and requirement for ctDNA detection pre-operatively will have affected the pre-test probability of post-operative ctDNA detection. This review will also have been subject to methodological heterogeneity due to the range of assays used for ctDNA analysis. Subgroup analysis was performed to partially overcome this. There remained significant heterogeneity in subgroup analysis, probably as a result of the large number of contributing variables. Of note, statistical testing demonstrated no appreciable heterogeneity within the metastasectomy subgroup, confirming disease stage to be one of the sources of heterogeneity.

Many of the studies in this review were small and exploratory in nature. There was no minimum sample size for inclusion, resulting in the inclusion of a few studies with small numbers of patients. However, for inclusion in the meta-analysis there had to be sufficient participants for survival analysis calculation to be performed. Quality assessment looked at the likelihood of bias due to differences in the management of ctDNA-positive and -negative groups. For a ‘low bias’ score the treating clinicians had to be blinded to the ctDNA results, which was the case in 15 studies. A further significant source of bias would be confounding due to the effects of adjuvant chemotherapy with only 12 studies outlining the proportion of participants who received adjuvant chemotherapy. Overall, it was felt that bias due to the classification of interventions and measurement outcomes was low.

Funnel plot asymmetry was observed, suggestive of publication bias. This is likely due to inclusion of a number of smaller studies and was partly overcome by obtaining individual participant data where possible to calculate HRs. Whilst this might exaggerate the magnitude of effect, the fact that the association was consistently observed across the studies suggests a true relationship. In addition, sample size calculations were performed in four of the included studies, demonstrating that shorter PFS associated with ctDNA detection reaches statistical significance when suitably powered [29, 31, 37, 47]. Large scale observational trials are already underway to establish the prognostic implications of ctDNA detection following surgery. Preliminary results from the GALAXY trial demonstrated a significantly shorter PFS with ctDNA detection at both 4 and 12 weeks post-op, and a higher rate of ctDNA clearance with adjuvant chemotherapy [48]. Interventional trials are also underway investigating the effectiveness of ctDNA in directing adjuvant chemotherapy use [49] and recent results from the DYNAMIC trial demonstrated non-inferiority with ctDNA guided selection to adjuvant chemotherapy [50].

A further limitation of this review was the inclusion of participants with incomplete surgical resections within some of the studies, which would preclude the analysis of MRD. Inclusion of studies that did not test for matched germline mutations may have resulted in false positives due to CHIP. Patients who had undergone curative treatment by other modalities such as chemoradiotherapy were not included, as this was outside the scope of this review.

## Conclusions

To conclude, ctDNA detection after curative surgery for colorectal cancer is a marker of poor prognosis. Here we demonstrate for the first time via meta-analysis that ctDNA detection post-operatively is associated with a significantly shorter PFS. Despite this wide body of evidence, there remains no consensus on many logistical aspects, most notably in the timing and method of analysis resulting in the considerable heterogeneity of this review and remains the greatest limitation to the clinical utility of this phenomenon.

## Supplementary information

Supplementary Material Faulkner et al.

Reporting Summary checklist

## Data Availability

All data supporting the findings of this study are available within the article and Supplementary Files.
