# Supplementary material for: The utility of ctDNA in detecting minimal residual disease following curative surgery in colorectal cancer: a systematic review and meta-analysis
Source: Br J Cancer. 2022 Nov 8;128(2):297–309. doi: 10.1038/s41416-022-02017-9 (PMC9902552; doi:10.1038/s41416-022-02017-9)
Supplement: Supplementary file 1 — Supplementary Material Faulkner et al. [file 41416_2022_2017_MOESM1_ESM.pdf]

Supplementary Material

Supplementary Figures

Supplementary Figure 1. Multivariate Analysis
Supplementary Figure 2. Overall survival

Supplementary Tables

Supplementary Table 1. Quality Assessment domains.
Supplementary Table 2. Key excluded studies.
Supplementary Table 3. Overall Survival.
Supplementary Table 4. Quality Assessment.

Supplementary Material

Search strategies
Data Extraction form
Quality Assessment form

References

Study or Subgroup	log[Hazard Ratio]	SE	Weight	Hazard Ratio IV, Random, 95% CI
Boysen 2020	2.0122	0.8301	5.5%	7.48 [1.47, 38.06]
Chen 2021	2.0819	0.4101	8.8%	8.02 [3.59, 17.92]
Murahashi 2020	2.0412	0.8017	5.7%	7.70 [1.60, 37.06]
Murray 2018	1.3376	0.4756	8.2%	3.81 [1.50, 9.68]
Reinert 2019	1.5041	0.5276	7.8%	4.50 [1.60, 12.66]
Ryan 2003	1.8516	0.5287	7.8%	6.37 [2.26, 17.95]
Taieb 2021	0.4383	0.1612	10.5%	1.55 [1.13, 2.13]
Tarazona 2019	2.4544	0.5889	7.3%	11.64 [3.67, 36.92]
Tie 2016	2.6391	0.3684	9.1%	14.00 [6.80, 28.82]
Tie 2019 (a)	2.0149	0.3889	8.9%	7.50 [3.50, 16.07]
Tie 2019 (b)	1.7918	0.5119	7.9%	6.00 [2.20, 16.36]
Tie 2021	1.141	0.5822	7.3%	3.13 [1.00, 9.80]
Zhong 2020	1.8221	0.8628	5.3%	6.18 [1.14, 33.55]
**Total (95% CI)**			**100.0%**	**5.73 [3.34, 9.84]**

Heterogeneity: Tau² = 0.70; Chi² = 56.70, df = 12 (P < 0.00001); I² = 79%
Test for overall effect: Z = 6.32 (P < 0.00001)

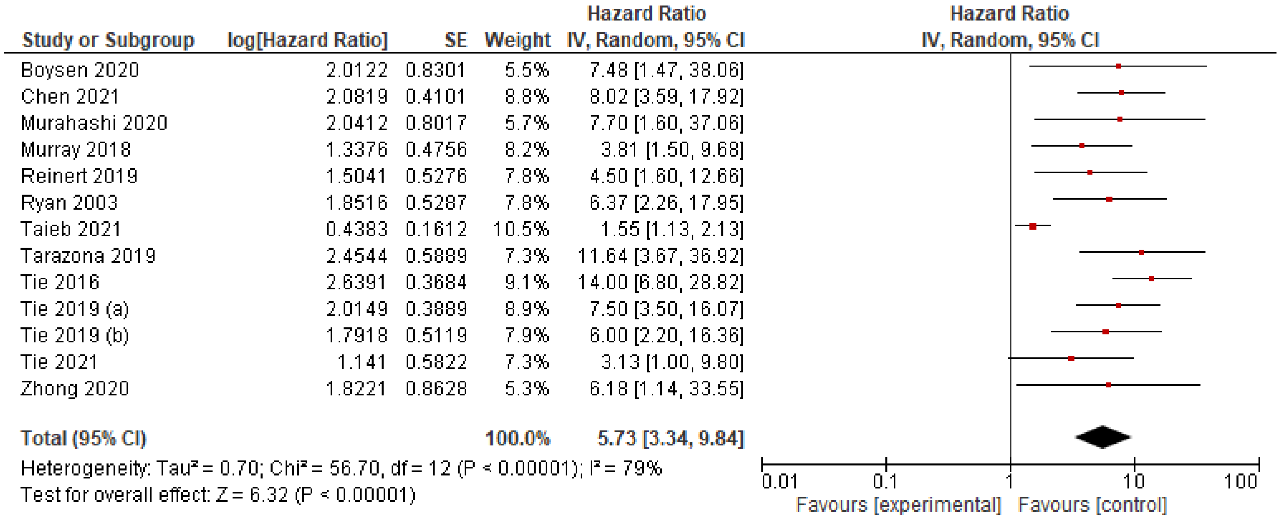

Supplementary Figure 1. Forest plot showing meta-analysis for PFS according to post-operative ctDNA in patients undergoing curative surgical resection for colorectal cancer where multivariate analysis was performed. Data displayed as HR with 95% confidence intervals on a logarithmic scale. HR- hazard ratio PFS- progression-free survival SE-standard error.

Study or Subgroup	log[Hazard Ratio]	SE	Weight	Hazard Ratio IV, Random, 95% CI
Khakoo 2020	1.8083	1.3901	7.0%	6.10 [0.40, 93.02]
Murray 2018	1.8871	0.6353	19.4%	6.60 [1.90, 22.93]
Scholer 2017 (I)	1.9021	0.7307	16.9%	6.70 [1.60, 28.06]
Taieb 2021	0.4447	0.1876	33.9%	1.56 [1.08, 2.25]
Tie 2021	1.4351	0.5253	22.8%	4.20 [1.50, 11.76]
**Total (95% CI)**			**100.0%**	**3.64 [1.63, 8.12]**

Heterogeneity: $Tau^2 = 0.46$; $Chi^2 = 10.61$, df = 4 (P = 0.03); $I^2 = 62\%$
Test for overall effect: Z = 3.16 (P = 0.002)

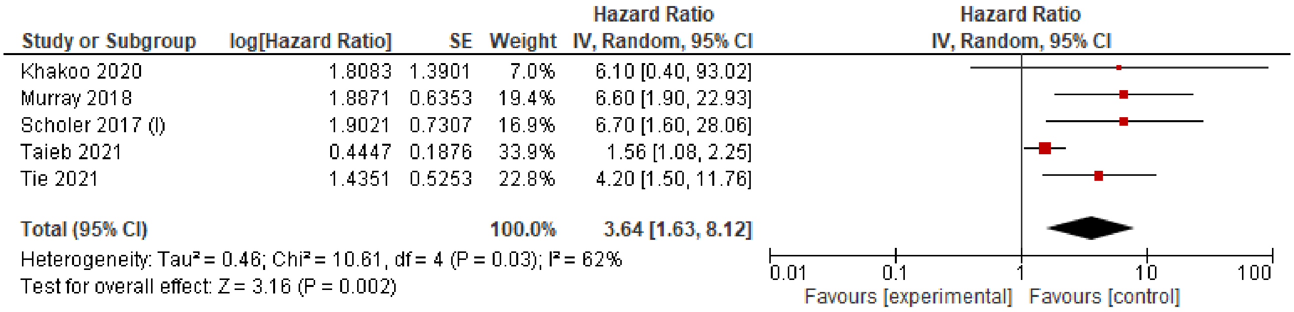

Supplementary Figure 2. Forest plot showing meta-analysis for OS according to post-operative ctDNA in patients undergoing curative surgical resection for colorectal cancer. Data displayed as HR with 95% confidence intervals on a logarithmic scale. HR- hazard ratio OS-overall survival SE-standard error.

**Pre-intervention**	
Bias due to confounding	Q2, Q4, Q5, Q10
Bias in the selection of participants into the study	Q2, timeline (collected in data extraction)
**At intervention**	
Bias in the classification of interventions	Q6, Q7, Q8
**Post-intervention**	
Bias due to deviation from intended interventions	Q5
Bias due to missing data	Q9
Bias in the measurement outcomes	Q12
Bias in the selection of the reported results	Q13

Supplementary table 1. Quality Assessment domains. Table to show the mapping of the Quality Assessment proforma used to the domains of bias of the ROBINS-I tool.

Author	Year	Reason for exclusion	Reference
Appelt	2020	Outcomes reported according to pre-operative ctDNA and ctDNA was not measured after surgery	(1)
Baumgartner	2018	Outcomes reported according to pre-operative ctDNA and ctDNA was not measured after surgery	(2)
Bazan	2006	Outcomes reported according to pre-operative ctDNA and ctDNA was not measured after surgery	(3)
Bidard	2019	Outcomes reported according to pre-operative ctDNA and ctDNA was not measured after surgery	(3)
Boysen	2017	Outcomes reported according to pre-operative ctDNA and ctDNA was not measured after surgery	(4)
Cassinotti	2013	Does not report the proportion of patients with disease relapse, do not report the proportion of patients classified as ctDNA positive after surgery	(5)
Frattini	2006	Outcomes not presented in terms of ctDNA after surgery	(6)
Frattini	2008	Does not give long-term outcomes according to post surgery ctDNA, authors contacted no reply	(7)
Garcia-Olmo	2012	Does not give PFS according to post surgery ctDNA	(8)
Hao	2014	Unable to differentiate surgical patients from non-surgical patients, disease relapse not reported	(9)
Hendricks	2018	Measures CTC not ctDNA	(10)
Herbst	2009	Outcomes reported according to pre-operative ctDNA and ctDNA was not measured after surgery	(11)
Hsieh	2005	Outcomes reported according to pre-operative ctDNA and ctDNA was not measured after surgery	(12)
Iwai	2019	Measures cfDNA not ctDNA	(13)
Li	2020	Only a single patient in their sample underwent surgery for CRC	(14)
Lin	2014	Outcomes reported according to pre-operative ctDNA and ctDNA was not measured after surgery	(15)
Narayan	2019	Outcomes reported according to pre-operative ctDNA and ctDNA was not measured after surgery	(16)
Pazdirek	2020	Outcomes reported according to pre-operative ctDNA and ctDNA was not measured after surgery	(17)
Polivka	2020	Outcomes not presented in terms of ctDNA after surgery	(18)
Schou	2018	Measures cfDNA not ctDNA	(19)
Symonds	2018	Patients not followed up after surgery	(20)
Vidal	2021	Outcomes reported according to pre-operative ctDNA and ctDNA was not measured after surgery	(21)
Vietsch	2018	Wrong study population: retrospectively selected patients who had disease relapse	(22)
Wang	2004	Outcomes not presented in terms of ctDNA after surgery	(23)
Wang	2019	Outcomes presented in terms of ctDNA status during follow-up rather than post-operatively	(24)
Wong	2019	ctDNA analysis in the cohort was not performed routinely post-surgery but at time of concern of disease progression from CT, does not address the issue of MRD	(25)
Yeh	2020	Disease relapse and survival not measured during the follow-up period	(26)
Zhu	2021	Measures cfDNA not ctDNA, no long term follow-up	(27)
Zhong	2020	Measures cfDNA not ctDNA	(28)

Supplementary Table 2. Key excluded studies. Table to show the main excluded studies and reason for exclusion from the review. cfDNA- cell free DNA CTC- circulating tumour cells ctDNA- circulating tumour DNA CRC- colorectal cancer MRD- minimal residual disease

Study ID	Median Survival (months)		Univariate			Multivariate		
	ctDNA positive	ctDNA negative	Hazard ratio	Confidence interval	p value	Hazard ratio	Confidence interval	p value
He 2020	not reached	not reached	not stated	not stated	not stated	-	-	-
Ji 2021	not reached	not reached	not stated	not stated	0.529	-	-	-
Khakoo 2020	not stated	not stated	6.1	0.4-97.1	0.2	-	-	-
López-Rojo 2020	22.8	35.4	-	-	-	-	-	-
Mason 2021 (>4 mutations)	[41% at 2 years]	[96% at 2 years]	not stated	not stated	<0.001	6.60	2.07-21.06	0.001
Murray 2018	Not stated	Not stated	6.6	1.9-22.8	0.011	-	-	-
Scøhler 2017 **(**Primary resection)	Not stated	Not stated	6.7	1.6-28.7	0.01	-	-	-
Taieb 2021	[81% at 5 years]	[87% at 5 years]	1.56	1.08-2.26	0.018	1.65	1.12-2.43	0.0108
Tie 2021	[31.7% at 5 years]	[77.7% at 5 years]	4.2	1.5-11.8	<0.001	-	-	-

Supplementary Table 3. Overall Survival. Table to show time-to-death following surgery according to post surgery ctDNA status. Confidence intervals displayed as 95%CI. CI- confidence interval. HR- hazard ratio.

Study ID	Centre number	Power calculations	Adjustment	Patient population	Co-interventions	Management	ctDNA methodology	Cut-off value	Timeline consistency	Attrition	Blinding	Relapse definition	Selective reporting	Timeline	Attrition (%)	Total
Allegretti 2020	Single	Nil	Nil	High	High	Low	Low	Low	Low	Low	Low	Low	High	Prospective	16.7	8
Beagan 2020	Single	Nil	Nil	Low	High	Low	Low	Low	High	Low	High	Low	Low	Prospective	0	7
Benešová 2019	Multicentre	Nil	Nil	High	High	Low	Low	Low	Low	Low	High	High	Low	Prospective	7.1	8
Boysen 2020	Single	Nil	Performed	Low	Low	Low	Low	Low	Low	Low	High	High	Low	Prospective	0	8
Carpinetti 2015	Single	Nil	Nil	High	Low	Low	High	Low	High	Low	Low	Low	Low	Prospective	0	8
Chen 2021	Multicentre	Nil	Performed	Low	Low	Low	Low	Low	Low	Low	Low	Low	Low	Prospective	3.6	11
Diehl 2008	Single	Nil	Nil	Low	Low	Low	Low	Low	Low	Low	Low	Low	Low	Prospective	0	11
He 2020	Single	Nil	Nil	Low	Low	Low	Low	Low	Low	Low	High	High	Low	Prospective	5	9
Huang 2019	Single	Nil	Nil	Low	Low	Low	Low	Low	Low	Low	Low	High	Low	Prospective	4.7	11
Ji 2021	Single	Nil	Nil	Low	High	Low	Low	Low	Low	High	High	High	High	Retrospective study design	30.4	6
Jin 2021	Single	Nil	Nil	Low	Low	Low	Low	Low	Low	Low	High	High	Low	Prospective	0	9
Khakoo 2020	Single	Nil	Nil	Low	Low	Low	Low	Low	High	Low	High	High	High	Prospective	0	7
Lee 2021	Single	Nil	Nil	Low	High	Low	Low	Low	Low	Low	Low	High	Low	Prospective	0	9
LeonArellano 2020	Single	Nil	Nil	Low	High	Low	Low	Low	Low	Low	Low	Low	Low	Prospective	0	10
Levy 2012	Single	Nil	Nil	Low	Low	Low	Low	High	Low	Low	High	Low	Low	Prospective	0	9
Lindforss 2005	Single	Nil	Nil	Low	High	Low	Low	High	Low	Low	High	High	Low	Prospective	0	7
López-Rojo 2020	Single	Nil	Nil	Low	Low	Low	Low	Low	Low	Low	High	High	Low	Prospective	4	9
Mason 2021	Single	Nil	Performed	Low	Low	High	Low	Low	High	Low	High	Low	High	Prospective	18.2	7
Murahashi 2020	Single	Nil	Performed	Low	High	Low	Low	Low	Low	High	High	High	Low	Prospective	0	7
Murray 2018	Single	Performed	Performed	Low	High	Low	Low	Low	High	Low	High	Low	Low	Prospective	23.4	8
Ng 2017	Single	Nil	Nil	Low	High	Low	Low	Low	Low	Low	High	High	Low	Prospective	0	8
Parikh 2021	Single	Nil	Performed	Low	Low	Low	Low	Low	High	Low	Low	Low	Low	Prospective	16.7	10

Reinert 2019	Multicentre	Nil	Performed	Low	Low	Low	Low	Low	Low	Low	Low	Low	Low	Prospective	16. 7	11
Ryan 2003	Single	Nil	Performed	Low	Low	Low	Low	Low	Low	Low	High	High	Low	Prospective	3.8	10
Schøler 2017	Single	Nil	Nil	Low	Low	Low	Low	Low	Low	Low	High	Low	High	Retrospective design	Not stated	8
Suzuki 2020	Multicentre	Nil	Nil	Low	Low	Low	Low	Low	Low	Low	High	High	Low	Prospective	0	10
Taieb 2021	Low	Nil	Performed	Low	Low	Low	Low	Low	Low	Low	High	Low	Low	Retrospective	0	9
Tanaka 2021	Single	Nil	Nil	Low	Low	Low	Low	Low	Low	Low	High	Low	Low	Prospective	0	10
Tarazona 2019	Single	Nil	Performed	Low	Low	Low	Low	Low	Low	High	Low	Low	Low	Prospective	26.6	10
Tie 2016	Multicentre	Performed	Performed	Low	Low	Low	Low	Low	High	Low	Low	Low	Low	Prospective	8.7	10
Tie 2019 (a)	Low	Nil	Performed	Low	Low	Low	Low	Low	High	Low	Low	Low	Low	Prospective	0	10
Tie 2019 (b)	Multicentre	Performed	Performed	Low	Low	Low	Low	Low	High	Low	Low	High	Low	Prospective	0	9
Tie 2021	Low	Performed	Performed	Low	Low	Low	Low	Low	High	Low	Low	High	Low	Prospective	0	9
Yamada 2016	Single	Nil	Nil	Low	Low	Low	Low	Low	High	Low	High	High	Low	Prospective	0	8
Zhong 2020	Single	Nil	Performed	Low	High	Low	Low	Low	Low	Low	High	High	High	Prospective	0	7
Zhou 2016	Single	Nil	Nil	Low	Low	Low	Low	Low	Low	High	High	Low	High	Prospective	16.7	8
Zhou 2021	Multicentre	Nil	Nil	Low	Low	Low	Low	Low	High	Low	Low	High	Low	Prospective	8.2	9
Zou 2020	Single	Nil	Nil	Low	High	Low	Low	Low	Low	Low	High	Low	High	Prospective	3.4	8

Supplementary Table 4. Quality Assessment. Overall quality assessment score and breakdown of scores within domains for included studies and percentage of sample lost to attrition.

Search strategies
Embase

Database: Embase <1974 to 2021 July 08>

Search Strategy:

----

1 ("circulating tumo?r*" adj2 DNA).tw. (5593)

2 ("cell-free" adj2 DNA).tw. (10146)

3 ("circulating free" adj2 DNA).tw. (597)

4 (ctDNA or "ct DNA").tw. (7444)

5 (cfDNA or "cf DNA").tw. (5851)

6 (cftDNA or "cft DNA").tw. (41)

7 plasma DNA.tw. (1779)

8 serum DNA.tw. (656)

9 "circulating nucleic acid*".tw. (668)

10 (KRAS adj2 (mutat* or gene*)).tw. (15519)

11 "kirsten rat sarcoma viral oncogene".tw. (620)

12 "v-Ki-ras*".tw. (338)

13 "Ki-ras".tw. (1407)

14 "K-ras".tw. (9131)

15 (tp53 adj2 (mutat* or gene*)).tw. (15739)

16 "micro-satellite instability-high".tw. (2)

17 "microsatellite instability-high".tw. (802)

18 MSI-H.tw. (2778)

19 "liquid biops*".tw. (7495)

20 circulating tumor DNA/ (5288)

21 microsatellite instability/ (15445)

22 oncogene k ras/ (11316)

23 or/1-22 (83300)

24 (colorectal adj3 (cancer* or neoplasm* or malignan* or carcinoma* or tumo?r* or adenocarcinoma*)).tw. (201238)

25 (colon* adj3 (cancer* or neoplasm* or malignan* or carcinoma* or tumo?r* or adenocarcinoma*)).tw. (129865)

26 (bowel* adj3 (cancer* or neoplasm* or malignan* or carcinoma* or tumo?r* or adenocarcinoma*)).tw. (11500)

27 (rectal* adj3 (cancer* or neoplasm* or malignan* or carcinoma* or tumo?r* or adenocarcinoma*)).tw. (50418)

28 (rectum adj3 (cancer* or neoplasm* or malignan* or carcinoma* or tumo?r* or adenocarcinoma*)).tw. (7573)

29 (sigmoid adj3 (cancer* or neoplasm* or malignan* or carcinoma* or tumo?r* or adenocarcinoma*)).tw. (3575)

30 CRC.tw. (59576)

31 exp colon cancer/ (291109)

32 rectum cancer/ (36860)

33 colon tumor/ (21603)

34 large intestine tumor/ (197)

35 colorectal tumor/ (25343)

36 rectum tumor/ (13637)

37 or/24-36 (439940)

38 colectom*.tw. (21560)

39 proctectom*.tw. (2347)

40 proctocolectom*.tw. (4695)

41 metastectom*.tw. (232)

42 exp abdominal surgery/ (827602)

43 exp intestine surgery/ (222175)

44 surg*.ti. (765246)

45 postsurg*.tw. (26667)

46 postoperat*.tw. (758237)

47 resect*.ti. (118531)

48 su.fs. (2129556)

49 or/38-48 (3290004)

50 23 and 37 and 49 (2794)

****

Medline

Database: Ovid MEDLINE(R) ALL <1946 to July 08, 2021>

Search Strategy:

----

1 ("circulating tumo?r*" adj2 DNA).tw. (2634)

2 ("cell-free" adj2 DNA).tw. (5594)

3 ("circulating free" adj2 DNA).tw. (282)

4 (ctDNA or "ct DNA").tw. (4031)

5 (cfDNA or "cf DNA").tw. (2532)

6 (cftDNA or "cft DNA").tw. (14)

7 plasma DNA.tw. (1032)

8 serum DNA.tw. (490)

9 "circulating nucleic acid*".tw. (300)

10 (KRAS adj2 (mutat* or gene*)).tw. (7132)

11 "kirsten rat sarcoma viral oncogene".tw. (543)

12 "v-Ki-ras*".tw. (312)

13 "Ki-ras".tw. (1364)

14 "K-ras".tw. (6327)

15 (tp53 adj2 (mutat* or gene*)).tw. (8031)

16 "micro-satellite instability-high".tw. (1)

17 "microsatellite instability-high".tw. (458)

18 MSI-H.tw. (1444)

19 "liquid biops*".tw. (4157)

20 Circulating Tumor DNA/ (1377)

21 or/1-20 (37572)

22 (colorectal adj3 (cancer* or neoplasm* or malignan* or carcinoma* or tumo?r* or adenocarcinoma*)).tw. (134187)

23 (colon* adj3 (cancer* or neoplasm* or malignan* or carcinoma* or tumo?r* or adenocarcinoma*)).tw. (93329)

24 (bowel* adj3 (cancer* or neoplasm* or malignan* or carcinoma* or tumo?r* or adenocarcinoma*)).tw. (7177)

25 (rectal* adj3 (cancer* or neoplasm* or malignan* or carcinoma* or tumo?r* or adenocarcinoma*)).tw. (32836)

26 (rectum adj3 (cancer* or neoplasm* or malignan* or carcinoma* or tumo?r* or adenocarcinoma*)).tw. (6422)

27 (sigmoid adj3 (cancer* or neoplasm* or malignan* or carcinoma* or tumo?r* or adenocarcinoma*)).tw. (2747)

28 CRC.tw. (36057)

29 exp Colorectal Neoplasms/ (211959)

30 or/22-29 (295525)

31 colectom*.tw. (12740)

32 proctectom*.tw. (1350)

33 proctocolectom*.tw. (3136)

34 metastectom*.tw. (88)

35 Colorectal Surgery/ (3719)

36 exp Digestive System Surgical Procedures/ (383491)

37 surg*.tw. (2037096)

38 postsurg*.tw. (20945)

39 postoperat*.tw. (572414)

40 resect*.tw. (374680)

41 su.fs. (2072247)

42 or/31-41 (3472737)

43 21 and 30 and 42 (1310)

****

Cochrane

Search Name: ctDNA and colorectal cancer

Date Run: 09/07/2021 20:01:09

Comment: 090721

ID	Search	Hits
#1	((circulating NEXT tumo?r*) NEAR/2 DNA):ti,ab	416
#2	(cell-free NEAR/2 DNA):ti,ab	226
#3	("circulating free" NEAR/2 DNA):ti,ab	28
#4	(ctDNA OR "ct DNA"):ti,ab	454
#5	(cfDNA OR "cf DNA"):ti,ab	164
#6	(cftDNA OR "cft DNA"):ti,ab	2
#7	"plasma DNA":ti,ab	55
#8	"serum DNA":ti,ab	19
#9	("circulating nucleic" NEAR/2 acid*):ti,ab	5
#10	(KRAS NEAR/2 (mutat* OR gene*)):ti,ab	663
#11	"kirsten rat sarcoma viral oncogene":ti,ab	40
#12	v-Ki-ras*:ti,ab	15
#13	Ki-ras:ti,ab	5
#14	K-ras:ti,ab	231
#15	(tp53 NEAR/2 (mutat* OR gene*)):ti,ab	412
#16	"micro-satellite instability-high":ti,ab	0
#17	"microsatellite instability-high":ti,ab	55
#18	MSI-H:ti,ab	99
#19	("liquid" NEAR/2 biops*):ti,ab	128
#20	[mh ^"Circulating Tumor DNA"]	16
#21	{OR #1-#20}	2251
#22	(colorectal NEAR/3 (cancer* OR neoplasm* OR malignan* OR carcinoma* OR tumo?r* OR adenocarcinoma*)):ti,ab	14177
#23	(colon* NEAR/3 (cancer* OR neoplasm* OR malignan* OR carcinoma* OR tumo?r* OR adenocarcinoma*)):ti,ab	4584
#24	(bowel* NEAR/3 (cancer* OR neoplasm* OR malignan* OR carcinoma* OR tumo?r* OR adenocarcinoma*)):ti,ab	629
#25	(rectal* NEAR/3 (cancer* OR neoplasm* OR malignan* OR carcinoma* OR tumo?r* OR adenocarcinoma*)):ti,ab	3737

#26 (rectum NEAR/3 (cancer* OR neoplasm* OR malignan* OR carcinoma* OR tumo?r* OR adenocarcinoma*)):ti,ab 587

#27 (sigmoid NEAR/3 (cancer* OR neoplasm* OR malignan* OR carcinoma* OR tumo?r* OR adenocarcinoma*)):ti,ab 89

#28 CRC:ti,ab 4461

#29 [mh "Colorectal Neoplasms"] 8554

#30 {OR #22-#29} 22347

#31 colectom*:ti,ab 1097

#32 proctectom*:ti,ab 74

#33 proctocolectom*:ti,ab 138

#34 metastectom*:ti,ab 9

#35 [mh ^"Colorectal Surgery"] 202

#36 [mh "Digestive System Surgical Procedures"] 14341

#37 surg*:ti,ab 212530

#38 postsurg*:ti,ab 6549

#39 postoperat*:ti,ab 112538

#40 resect*:ti,ab 26960

#41 MeSH descriptor: [] explode all trees and with qualifier(s): [surgery - SU] 59936

#42 {OR #31-#41} 276555

#43 #21 AND #30 AND #42 196

Quality Assessment form

1. Single or multicentre study
Number of centres patients were recruited from
Single- high risk mulit centre- low risk

2. Patient population
Are the patient population clearly described adequate baseline clinical data collected on cohort
high-no Low-yes

3. Sample size and power calculations
Number of patients recruited, sample size calculations
Low- sample size calculations performed High- not performed

4. Are any co intervention clearly and unambiguously stated
Did patients receive adjuvant chemotherapy following resection, if so was this accounted for and were outcomes presented separately in terms of this.
Did all patients receiving adjuvant chemotherapy receive the same therapy (regimes, duration)
Were the surgical resections performed equivalent between the two groups, were lymph node involvement outcomes comparable, did all patients in the cohort achieve R0 resections
High- no, Low- yes

5. Were different management strategies initiated based on ctDNA results
Were ctDNA levels used to guide treatment decisions
High- yes Low- no

6. ctDNA methodology
was the ctDNA methodology was adequately described
Low- adequately described. High- not well described

7. Cut-off value
Description of cut off for consideration of ctDNA positive/detectable, was this defined beforehand ctDNA status defined prospectively at the time of measurement and not retrospectively after the follow up period
low- clearly defined high- ambiguous

8. Time line consistency
Were there time line consistencies for all ctDNA measurements, how wide was the timing interval over which measurements could be taken
low- set timeframe stated in methods samples collected from sample at same time following surgerry, high- wide range

9. Attrition
Are attrition rates adequately described
high- well described low - poorly described

10. Adjustment
Was multi variate analysis performed, were outcomes presented adjusted for cofounding variables
high- no low- yes

11. Blinding
Were the treating physicians blinded to the ctDNA results when making treatment decisions (ie decisions regarding adjuvant chemotherapy), were assessors blinded to the ctDNA result (positive/negative) when collecting follow up data (OS, PFS)
high- no blinding, low- blinded

12. Clear definition of recurrence
Definition of recurrence- was it confirmed radiologically, was the time at radiological confirmation of recurrence used in calculating PFS.
high- not clearly defined, low- clearly defined, radiologically

13. Selective reporting of results
Outcomes for all groups clearly presented
yes- low, no-high

Data Extraction form

**1. Study Details**
Study ID
Covidence ID
Title
Author<br>*Surname of first author et al*
Journal published in
Year of publication
Country<br>*Country in which patients were treated*
Journal
Trial ID number or local ethics number
Study centre number<br>*Number of centres data collection took place in*
Additional notes
**2. Study Design**
Sample size<br>*Total number of patient who completed the study (addressing our study objective), Not including healthy controls or other study groups in the study addressing other research questions*
Attrition<br>*Total attrition number and attrition according to group (ctDNA positive vs negative). Attrition defined as withdrawal from study due to unexpected natural causes not part of disease process or outcomes, eg. lost to follow up, withdrew consent*
Number of patients completed all follow-up
Follow up duration<br>*Median follow up, in months*
Follow up - range<br>*Follow up range in months*
Study timeline<br>☐ Prospective<br>☐ Retrospective<br>☐ Other
Study aims: Primary and secondary study objectives
Inclusion criteria
Exclusion criteria
Post operative monitoring<br>*Method, frequency and duration of post op monitoring after surgery*
Additional notes
**3. Patient characteristics**
Age<br>*Median age of participant in years to the nearest year*<br>☐ Age statistic<br>☐ Median<br>☐ Mean<br>☐ Mode<br>☐ Not stated<br>☐ Other
Gender<br>*%male to one decimal place*
Histology<br>Histology of cancer primary<br>☐ Adenocarcinoma

- ☐ GIST
- ☐ NET
- ☐ Not stated
- ☐ Other

Cancer stage

Cancer site
- ☐ Colon
- ☐ Rectal
- ☐ Colon and rectal
- ☐ Metastases
- ☐ Other

Site- percentage colon
Percentage of cases where the primary site was colon (vs. rectal)

Cancer side
percentage right sided colon cancer to one decimal place. If not mentioned or not applicable 'not stated'

Additional notes

Chemotherapy

Neoadjuvant chemotherapy
Proportion of patients in cohort who had chemotherapy prior to surgery (% to one decimal place)

Neoadjuvant chemotherapy regimes

Adjuvant chemotherapy: percentage of patients
Proportion of patients in cohort who had chemotherapy prior to surgery (% to one decimal place)
Adjuvant chemotherapy regime

Adjuvant chemotherapy duration

Number of cycles

4. ctDNA measurement

Pre surgery levels
Was a liquid biopsy performed prior to surgery
- ☐ Yes
- ☐ No

Timing of pre surgery levels

Number of liquid biopsy post-surgery

Time range over which all liquid biopsies were performed
Time from surgery to timing of last liquid biopsy performed

Timing of first measurement post-surgery
Time in months after surgery the first liquid biopsy was performed after surgery

Sample type
- ☐ Plasma
- ☐ Serum
- ☐ Not stated
- ☐ Other

Method of detection

Gene panel/ target genes

Limit of detection

Cut off level
Cut of level for which measurements were considered 'ctDNA positive', if any above limit of detection

Additional notes

5. Outcomes

Percentage with detectable ctDNA
Proportion of cohort who were considered ctDNA positive at first liquid biopsy after surgery (% to one decimal place)

Percentage with detectable ctDNA post-operatively who experienced disease recurrence during the follow-up period<br>*Recurrence defined as radiological recurrence during the follow up period, if more than one liquid biopsy was performed post surgery, patients should be stratified according to the first ctDNA measurement taken after curative surgery or after completion of adjuvant chemotherapy, Percentage to one decimal place*
Percentage with undetectable ctDNA post operatively who experienced disease recurrence during the follow-up period<br>*Recurrence defined as radiological recurrence during the follow up period, if more than one liquid biopsy was performed post surgery, patients should be stratified according to the first ctDNA measurement taken after curative surgery or after completion of adjuvant chemotherapy, percentage to one decimal place*
Mutations detected<br>*Main genes in which mutations were seen*
Percentage with mutations detected<br>*Percentage of patients in whom mutations were detected on ctDNA analysis*
Study conclusions
Multivariate analysis<br>*Were any adjustments for cofounding variables carried out, which variables were adjusted for*
Outcomes
Median PFS ctDNA positive<br>*Median PFS of ctDNA positive patients in months, to nearest month, for all patients*
Median PFS ctDNA negative<br>*Median PFS of ctDNA negative patients in months, to nearest month, for all patients in the cohort*
Hazard ratio<br>*Hazard ratio of PFS for ctDNA positive compared to negative for all patients in the cohort, with 95% confidence intervals and p value.*
Median PFS ctDNA positive (adjuvant chemotherapy)<br>*Median PFS of ctDNA positive patients in months, to nearest month, for patients who received adjuvant chemotherapy*
Median PFS ctDNA negative (adjuvant chemotherapy)<br>*Median PFS of ctDNA negative patients in months, to nearest month, for patients who received adjuvant chemotherapy*
Hazard ratio (adjuvant chemotherapy)<br>*Hazard ratio of PFS for ctDNA positive compared to negative for patients who received adjuvant chemotherapy, with 95% confidence intervals and p value*
Median PFS ctDNA positive (no adjuvant chemotherapy)<br>*Median PFS of ctDNA positive patients in months, to nearest month, for patients who did not receive adjuvant chemotherapy*
Median PFS ctDNA negative (no adjuvant chemotherapy)<br>*Median PFS of ctDNA negative patients in months, to nearest month, for patients who did not receive adjuvant chemotherapy*
Hazard ratio (no adjuvant chemotherapy)<br>*Hazard ratio of PFS for ctDNA positive compared to negative for patients who did not receive adjuvant chemotherapy, with 95% confidence intervals and p value*
Overall survival
Median survival ctDNA positive<br>*In months*
Median survival ctDNA negative<br>*In months*
Hazard ratio<br>*Hazard ratio of OS for ctDNA positive compared to negative patient, with 95% confidence intervals and p value*

References

1. Appelt AL, Andersen RF, Lindebjerg J, Jakobsen A. Prognostic Value of Serum NPY Hypermethylation in Neoadjuvant Chemoradiotherapy for Rectal Cancer. Am J Clin Oncol [Internet]. 2020;43(1):9–13.

2. Baumgartner JM, Raymond VM, Lanman RB, Tran L, Kelly KJ, Lowy AM, et al. Preoperative Circulating Tumor DNA in Patients with Peritoneal Carcinomatosis is an Independent Predictor of Progression-Free Survival. Ann Surg Oncol [Internet]. 2018 ;25(8):2400–8.

3. Bazan V, Bruno L, Augello C, Agnese V, Calo V, Corsale S, et al. Molecular detection of TP53, Ki-Ras and p16INK4A promoter methylation in plasma of patients with colorectal cancer and its association with prognosis. Results of a 3-year GOIM (Gruppo Oncologico dell'Italia Meridionale) prospective study. Ann Oncol Off J Eur Soc Med Oncol [Internet]. 2006 ;17 Suppl 7:vii84-90.

4. Bidard F-C, Kiavue N, Ychou M, Cabel L, Stern M-H, Madic J, et al. Circulating Tumor Cells and Circulating Tumor DNA Detection in Potentially Resectable Metastatic Colorectal Cancer: A Prospective Ancillary Study to the Unicancer Prodige-14 Trial. Cells. 2019;8(6).

5. Cassinotti E, Boni L, Segato S, Rausei S, Marzorati A, Rovera F, et al. Free circulating DNA as a biomarker of colorectal cancer. Int J Surg. 2013;11 Suppl 1:S54-7.

6. Frattini M, Gallino G, Signoroni S, Balestra D, Battaglia L, Sozzi G, et al. Quantitative analysis of plasma DNA in colorectal cancer patients: a novel prognostic tool. Ann N Y Acad Sci. 2006 ;1075:185–90.

7. Frattini M, Gallino G, Signoroni S, Balestra D, Lusa L, Battaglia L, et al. Quantitative and qualitative characterization of plasma DNA identifies primary and recurrent colorectal cancer. Cancer Lett. 2008;263(2):170–81.

8. García-Olmo D, García-Olmo DC, Domínguez-Berzosa C, Guadalajara H, Vega L, García-Arranz M. Oncogenic transformation induced by cell-free nucleic acids circulating in plasma (genometastasis) remains after the surgical resection of the primary tumor: A pilot study. Expert Opin Biol Ther. 2012 ;12(SUPPL. 1).

9. Hao TB, Shi W, Shen XJ, Qi J, Wu XH, Wu Y, et al. Circulating cell-free DNA in serum as a biomarker for diagnosis and prognostic prediction of colorectal cancer. Br J Cancer [Internet]. 2014;111(8):1482–9.

10. Hendricks A, Eggebrecht G-L, Bernsmeier A, Geisen R, Dall K, Trauzold A, et al. Identifying patients with an unfavorable prognosis in early stages of colorectal carcinoma. Oncotarget. 2018 8a;9(44):27423–34.

11. Herbst A, Wallner M, Rahmig K, Stieber P, Crispin A, Lamerz R, et al. Methylation of helicase-like transcription factor in serum of patients with colorectal cancer is an independent predictor of disease recurrence. Eur J Gastroenterol Hepatol. 2009;21(5):565–9.

12. Hsieh J-S, Lin S-R, Chang M-Y, Chen F-M, Lu C-Y, Huang T-J, et al. APC, K-ras, and p53 gene mutations in colorectal cancer patients: correlation to clinicopathologic features and postoperative surveillance. Am Surg. 2005;71(4):336–43.

13. Iwai T, Yamada T, Takahashi G, Takeda K, Koizumi M, Shinji S, et al. Circulating cell-free long DNA fragments predict post-hepatectomy recurrence of colorectal liver metastases. Eur J Surg Oncol. 2019;46(1):108–14.

14. Li J, Jiang W, Wei J, Zhang J, Cai L, Luo M, et al. Patient specific circulating tumor DNA fingerprints to monitor treatment response across multiple tumors. J Transl Med. 2020;18(1):293.

15. Lin J-K, Lin P-C, Lin C-H, Jiang J-K, Yang S-H, Liang W-Y, et al. Clinical relevance of alterations in quantity and quality of plasma DNA in colorectal cancer patients: based on the mutation spectra detected in primary tumors. Ann Surg Oncol. 2014;21 Suppl 4:S680-6.

16. Narayan RR, Goldman DA, Gonen M, Reichel J, Huberman KH, Raj S, et al. Peripheral Circulating Tumor DNA Detection Predicts Poor Outcomes After Liver Resection for Metastatic Colorectal Cancer. Ann Surg Oncol. 2019;26(6):1824–32.

17. Pazdirek F, Minarik M, Benesova L, Halkova T, Belsanova B, Macek M, et al. Monitoring of Early Changes of Circulating Tumor DNA in the Plasma of Rectal Cancer Patients Receiving Neoadjuvant Concomitant Chemoradiotherapy: Evaluation for Prognosis and Prediction of Therapeutic Response. Front Oncol. 2020;10:1028.

18. Polivka J, Windrichova J, Pesta M, Houfkova K, Rezackova H, Macanova T, et al. The Level of Preoperative Plasma KRAS Mutations and CEA Predict Survival of Patients Undergoing Surgery for Colorectal Cancer Liver Metastases. Cancers (Basel). 2020;12(9).

19. Schou J V., Larsen FO, Sørensen BS, Abrantes R, Boysen AK, Johansen JS, et al. Circulating cell-free DNA as predictor of treatment failure after neoadjuvant chemo-radiotherapy before surgery in patients with locally advanced rectal cancer. Ann Oncol. 2018;29(3):610–5.

20. Symonds EL, Pedersen SK, Murray DH, Jedi M, Byrne SE, Rabbitt P, et al. Circulating tumour DNA for monitoring colorectal cancer-a prospective cohort study to assess relationship to tissue methylation, cancer characteristics and surgical resection. Clin Epigenetics. 2018;10:63.

21. Vidal J, Casadevall D, Bellosillo B, Pericay C, Garcia-Carbonero R, Losa F, et al. Clinical Impact of Presurgery Circulating Tumor DNA after Total Neoadjuvant Treatment in Locally Advanced Rectal Cancer: A Biomarker Study from the GEMCAD 1402 Trial. Clin Cancer Res. 2021;27(10):2890–8.

22. Vietsch EE, Graham GT, McCutcheon JN, Javaid A, Giaccone G, Marshall JL, et al. Reprint of: Circulating cell-free DNA mutation patterns in early and late stage colon and pancreatic cancer. Cancer Genet . 2018;228–229:131–42.

23. Wang J-Y, Hsieh J-S, Chang M-Y, Huang T-J, Chen F-M, Cheng T-L, et al. Molecular detection of APC, K- ras, and p53 mutations in the serum of colorectal cancer patients as circulating biomarkers. World J Surg. 2004;28(7):721–6.

24. Wang Y, Li L, Cohen JD, Kinde I, Ptak J, Popoli M, et al. Prognostic Potential of Circulating Tumor DNA Measurement in Postoperative Surveillance of Nonmetastatic Colorectal Cancer. JAMA Oncol. 2019;5(8):1118–23.

25. Wong R, Tie J, Lee M, Cohen J, Wang Y, Li L, et al. The potential role of circulating tumor DNA (ctDNA) in the further investigation of colorectal cancer patients with nonspecific findings on standard investigations. Int J Cancer. 201915a;145(2):540–7.

26. Yeh Y-M, Lin P-C, Lee C-T, Chen S-H, Lin B-W, Lin S-C, et al. Treatment monitoring of colorectal cancer by integrated analysis of plasma concentration and sequencing of circulating tumor DNA. Mol Cancer. 2020;19(1):150.

27. Zhu F, Ma J, Ru D, Wu N, Zhang Y, Li H, et al. Plasma DNA Integrity as a Prognostic

Biomarker for Colorectal Cancer Chemotherapy. J Oncol. 2021;2021:5569783.

28. Zhong Y, Zhou Q, Zhang Y, Zhou S, Zhang G, Jiang C, et al. Cell-free DNA as a biomarker for colorectal cancer: a retrospective analysis in patients before and after surgery. Cell Mol Biol (Noisy-le-grand). 2020;66(2):135–41.
